# Elucidating the proteomic pathways linking childhood body mass index with type 2 diabetes: a Mendelian randomization and recall-by-genotype study

**DOI:** 10.1093/ije/dyag165

**Published:** 2026-08-18

**Authors:** Siyu Chen, Yiwen Liang, Shan Luo, Jie Zheng, Satoshi Yoshiji, Grace M Power, Zikun Xu, Stephen Burgess, Benjamin John Cowling, Hugh Simon Lam, Albert Martin Li, Gabriel Matthew Leung, Catherine Mary Schooling, Shiu Lun Au Yeung

**Affiliations:** School of Public Health, Li Ka Shing Faculty of Medicine, The University of Hong Kong, Hong Kong Special Administrative Region, China; School of Public Health, Li Ka Shing Faculty of Medicine, The University of Hong Kong, Hong Kong Special Administrative Region, China; School of Public Health, Li Ka Shing Faculty of Medicine, The University of Hong Kong, Hong Kong Special Administrative Region, China; Department of Family Medicine and Primary Care, School of Clinical Medicine, Li Ka Shing Faculty of Medicine, The University of Hong Kong, Hong Kong Special Administrative Region, China; Department of Endocrine and Metabolic Diseases, Shanghai Institute of Endocrine and Metabolic Diseases, Ruijin Hospital, Shanghai Jiao Tong University School of Medicine, Shanghai, China; Department of Human Genetics, McGill University, Montréal, QC, Canada; MRC Integrative Epidemiology Unit, University of Bristol, Bristol, United Kingdom; Population Health Sciences, Bristol Medical School, University of Bristol, Bristol, United Kingdom; Institute for Molecular Bioscience, The University of Queensland, Brisbane, Queensland, Australia; Department of Electrical and Electronic Engineering, Imperial College London, London, United Kingdom; MRC Biostatistics Unit, Cambridge Institute of Public Health, University of Cambridge, Cambridge, United Kingdom; School of Public Health, Li Ka Shing Faculty of Medicine, The University of Hong Kong, Hong Kong Special Administrative Region, China; Department of Paediatrics, Faculty of Medicine, The Chinese University of Hong Kong, Hong Kong Special Administrative Region, China; Department of Paediatrics, Faculty of Medicine, The Chinese University of Hong Kong, Hong Kong Special Administrative Region, China; School of Public Health, Li Ka Shing Faculty of Medicine, The University of Hong Kong, Hong Kong Special Administrative Region, China; School of Public Health, Li Ka Shing Faculty of Medicine, The University of Hong Kong, Hong Kong Special Administrative Region, China; Graduate School of Public Health and Health Policy, City University of New York, New York, NY, United States; School of Public Health, Li Ka Shing Faculty of Medicine, The University of Hong Kong, Hong Kong Special Administrative Region, China

**Keywords:** childhood obesity, proteomics, type 2 diabetes

## Abstract

**Background:**

Increased childhood adiposity contributes to higher type 2 diabetes (T2D) risk, but the underlying mechanisms remain unclear. Furthermore, whether these mechanistic pathways are shared across ethnic groups has not been explored.

**Methods:**

We conducted a two-step Mendelian randomization (MR) using European-based summary statistics from genome-wide association studies of childhood body mass index (BMI) (*n *= 61 111), adulthood proteomics (*n *= 35 559), and later life T2D (cases: 242 283; controls: 1 569 734). We also conducted a recall-by-genotype (RbG) study in a Chinese birth cohort (*n *= 250) to assess whether childhood BMI was associated with the identified proteomic signals in an East Asian population. Analyses included multi-instrument MR methods (inverse variance weighted and MR-Egger) and, for single-instrument proteins, *cis*-MR using the Wald ratio, with colocalization validation. Multiple testing was adjusted using false discovery rate (FDR).

**Results:**

Higher genetically predicted childhood BMI was associated with 1299 proteins (FDR-adjusted *P *< .05), of which 36 proteins were associated with higher T2D risk. Six proteins were supported by colocalization (PP_H4_ > 80%). Genetically predicted higher childhood BMI was positively associated with GCKR, RBP1, ENTPD6, and GST A1-1, and inversely associated with PRSS27 and SHBG. The proportion of the BMI-T2D effect mediated by these proteins ranged from 1.87% to 12.92%. However, only SHBG was replicated in Asians in the RbG study.

**Conclusions:**

Our study identified putative actionable proteins that could reduce the elevated T2D risk attributable to childhood adiposity in Europeans, but their relevance in Asian populations requires further investigation.

Key Messages

- The detrimental effects of childhood adiposity on type 2 diabetes risk have been reported, but the underlying mechanisms, particularly the mediating proteins remain underexplored.
- In Europeans, genetically predicted childhood body mass index (BMI) was associated with type 2 diabetes risk partly through sex hormone-binding globulin (SHBG) and glucokinase regulatory protein (GCKR) (mediated proportions: approximately 2.8% and 13%); however, the proteomic signatures related to childhood BMI differed substantially in East Asians.
- Targeting these proteins could help to mitigate the rising burden of type 2 diabetes driven by childhood obesity, although further ethnic-specific studies are needed to assess generalizability.

## Introduction

Type 2 diabetes (T2D) is a major contributor to disease burden globally, accounting for 2.8% of total disability adjusted life years in 2019 [1]. While several factors are related to higher T2D risk, including poor sleep [2] and non-alcoholic fatty liver disease [3], the rising rate of childhood adiposity is increasingly recognized as a key driver of this epidemic [4], particularly in emerging economies such as China [5]. Although the detrimental effects of childhood adiposity on T2D risk have been reported using different epidemiologic designs [6], the underlying mechanisms remain underexplored beyond potential mediation through adult adiposity.

Proteomic studies can elucidate mechanistic pathways and inform therapeutic development. However, studies of childhood adiposity proteomic signatures (e.g. adipocytokines) have generally been small, leaving results vulnerable to bias [7]. To circumvent these limitations, Mendelian randomization (MR) studies have been increasingly used to assess mediation, given they are less susceptible to confounding through the random assortment of genetic variants at conception [8]. One main issue with earlier MR studies was the lack of ethnic-specific analyses, which can yield differing results by ethnicity [3, 9].

To comprehensively assess the mediating role of proteins in the relationship of childhood adiposity with T2D aetiology, we employed a two-step MR design using European-based summary statistics. We further explored the generalizability of our findings to East Asian populations using a recall-by-genotype (RbG) study in a Chinese birth cohort and East Asian-specific genome-wide association study (GWAS).

## Materials and methods

### Study design

**Figure 1:**
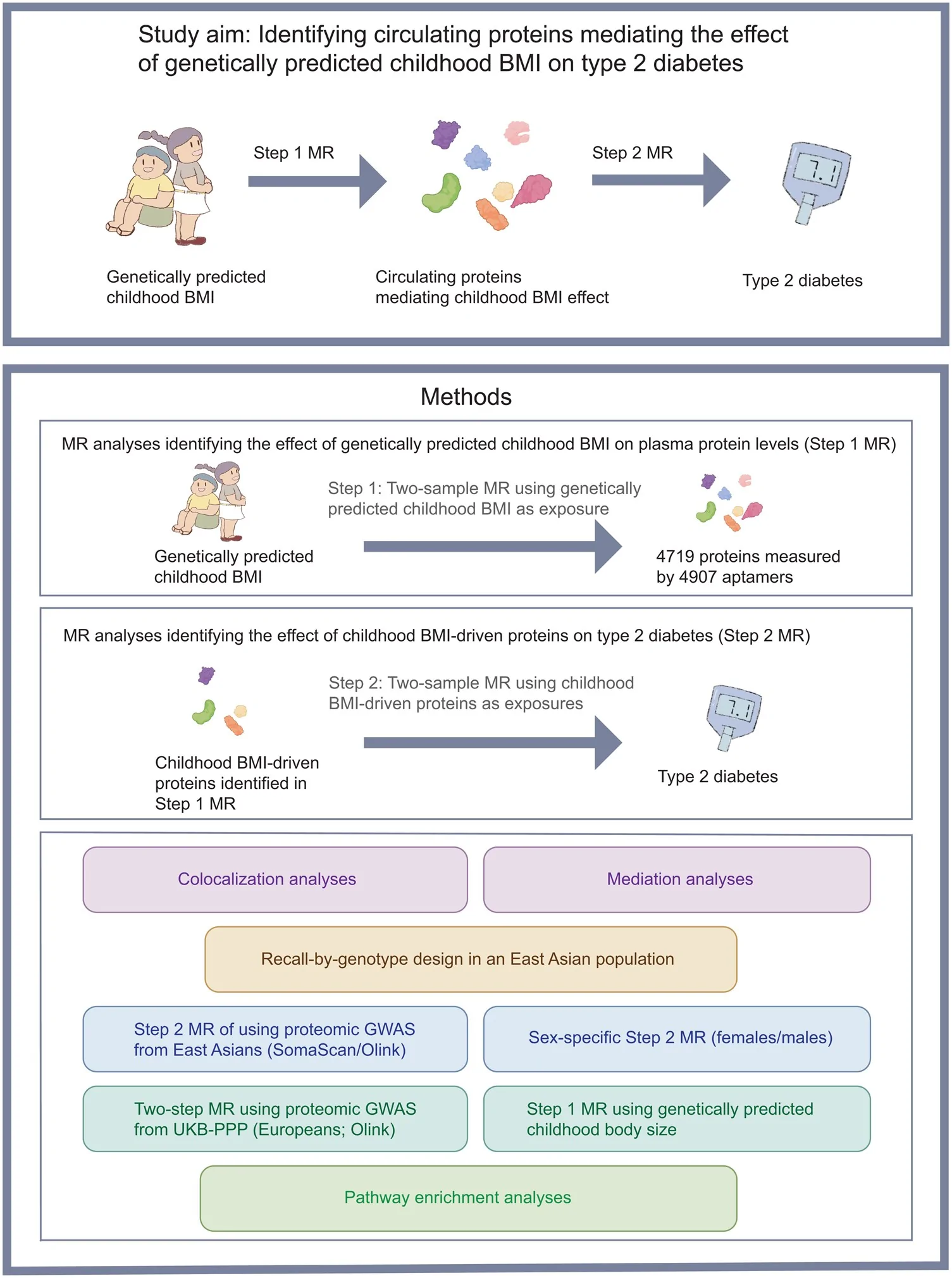
Schematic diagram of the study design. MR, Mendelian randomization; BMI, body mass index; GWAS, genome-wide association study; UKB-PPP, the UK Biobank Pharma Proteomics Project. Protein elements were generated using Biorender. Created in BioRender. Chen, S. (2026) https://BioRender.com/lu8gtx3 and https://BioRender.com/v23gy52.

This was a two-step MR study utilizing European-based summary statistics from GWAS (Fig. 1). In Step 1, we assessed the association of genetically predicted childhood adiposity (body mass index, BMI) with proteomic signatures. In Step 2, we assessed the associations of genetically predicted childhood BMI-related proteins with T2D risk. This study was reported in accordance with the Strengthening the Reporting of Observational Studies in Epidemiology using Mendelian Randomization (STROBE-MR) checklist [10].

### Instrumental variable assumptions

The three main instrumental variable assumptions of MR are: (i) Genetic instruments are associated with the exposure (relevance); (ii) Genetic variant-outcome association is not confounded (independence); and (iii) Genetic instruments influence the outcome only via the exposure (exclusion-restriction) [11].

### Genetic predictors of childhood BMI (exposure)

We extracted strong (*P *< 5 × 10^−8^) and independent (*r*^2^ < 0.001) instruments for childhood BMI from the Early Growth Genetics (EGG) Consortium, a GWAS including 61 111 European ancestry participants aged 2–10 years [12]. BMI was given as a sex- and age-adjusted standard deviation score at the latest time point (the oldest age if multiple measurements). The GWAS was adjusted for principal components where appropriate in individual studies before meta-analysis. We excluded variants in the human major histocompatibility (*MHC*) region (chr6:28 510 120–33 480 577) and the ABO blood group gene region (chr9: 133 174 055–133 504 070) to reduce the likelihood of horizontal pleiotropy.

### Genetic associations with proteins in adulthood

We used summary statistics from one of the largest protein GWAS, comprising 35 559 Icelanders (57% women, mean age 55 years). In total, 4719 plasma proteins were measured by 4907 aptamers using the SomaScan assay v4 (SomaLogic, Boulder, Colorado, United States) [13]. This platform was chosen for its high assay accuracy [14], and to maintain consistency with the proteomic platform used in our subsequent RbG design. Genetic associations were adjusted for age, sex, and sample age.

### Genetic predictors of adulthood proteins related to childhood BMI

Based on the findings from Step 1 MR, we extracted strong and independent *cis*-instruments for childhood BMI-driven proteins. We only considered *cis*-acting protein quantitative trait loci (*cis*-pQTLs) located within 1 Mb of transcription start site of protein-coding genes, as defined in the protein GWAS [13], to reduce the likelihood of horizontal pleiotropy [15].

### Genetic associations with type 2 diabetes (outcome)

Genetic associations with T2D were extracted from a GWAS of 242 283 cases and 1 569 734 controls of European ancestry [16]. The definitions of T2D varied across included studies (e.g. HbA1c, fasting glucose, self-reported physician-diagnosed diabetes, or use of diabetes medication). Associations were adjusted for age, sex, and study-specific covariates; population stratifications were accounted for using different approaches (e.g. adjusting for genetic relatedness index) [16].

Details of all included GWAS are in Supplementary Table S1.

### Statistical analyses

#### Step 1 MR (childhood BMI to proteins)

The main analysis used the inverse variance weighted (IVW) method with multiplicative random effects, which assumes balanced pleiotropy. We corrected for multiple comparisons (4907 aptamer-measured proteins) using false discovery rate (FDR-adjusted *P *< .05) [17]. Heterogeneity of the Wald ratios was assessed using Cochran’s *Q* test, where high heterogeneity indicates the presence of invalid instruments. F-statistic was approximated to assess instrument strength, where instruments with F-statistic < 10 were excluded to reduce weak instrument bias. The MR-Egger intercept was used to assess possible directional horizontal pleiotropy. We also included MR-Egger (assuming instrument strength independent of direct effect, InSIDE) and weighted median (where the majority of weights derive from valid instruments), which rely on different assumptions [18]. Proteins with directionally consistent estimates across IVW and weighted median were carried forward to Step 2 MR. Evidence against a causal effect of childhood BMI was considered when MR-Egger and IVW had inconsistent directions, and there was evidence of overall horizontal pleiotropy (MR-Egger intercept test *P *< .05), given that MR-Egger is less precise than IVW.

#### Step 2 MR (proteins to T2D risk)

IVW was used when a protein had more than one instrument; otherwise, the Wald ratio method was used. Because most proteins had few instruments (≤2), only Cochran’s *Q* was used to assess possible heterogeneity. To evaluate whether the identified proteins mediated the relation of childhood BMI with T2D, we checked that the directions of associations in Step 1 and Step 2 were consistent with the hypothesis that childhood BMI increases T2D risk before proceeding with downstream mediation analyses [19].

### Colocalization

For proteins identified in the MR analyses, we conducted genetic colocalization analyses to investigate whether the proteins and T2D shared a single causal variant within the *cis*-region of the protein-coding gene (±500kb around the *cis*-pQTL site). Posterior probability H4 (PP_H4_ > 80%) was considered strong evidence of a shared causal variant. We used default prior probabilities of *p*_1_ = *p*_2_ = 10^−4^ and *p*_12_ = 10^−5^, where *p*_1_ and *p*_2_ represent the prior probabilities that a specific variant is associated with either trait, and *p*_12_ denotes the prior probability that the same variant is associated with both traits [20]. To assess robustness, we tested various combinations of priors with stricter criteria [21], and applied SuSiE (Sum of Single Effects, with default priors) to shortlisted proteins to allow for multiple causal variants [22].

### Mediation analyses

For proteins supported by genetic colocalization, we estimated the proportion of the effect of childhood BMI on T2D risk mediated by each protein using the product-of-coefficients approach within the two-step MR framework, with standard errors calculated using the delta method [23].

### Recall-by-genotype design in the Chinese birth cohort

To explore whether childhood adiposity proteomic signals also present in East Asians, we implemented a recall-by-genotype design in a subset of the ‘Children of 1997’ birth cohort, a population-representative cohort in Hong Kong [24]. This design compares proteomic signals between extremes of a polygenic risk score (PRS) for childhood BMI, rather than extremes of childhood BMI itself, and is therefore more robust to confounding than conventional observational analyses [25]. We selected participants with the highest and lowest unweighted PRS of childhood BMI at age 10 (*n *= 250) based on 12 SNPs identified in a GWAS of childhood BMI within the same birth cohort (*n *= 3279, Supplementary Table S9). All SNPs were biallelic, had minor allele frequency (MAF) > 0.1%, *P *< 5 × 10^−6^, and were independent based on the 1000 Genome East Asian panel [26]. A total of 7289 plasma proteins were measured using SomaScan assay v4. Preliminary analyses showed no significant demographic differences between PRS groups (Supplementary Table S10); therefore, linear regression adjusted only for age and sex was used to compare protein levels between the highest and lowest PRS groups. Nominal significance (*P *< .05) was considered an indication of consistency with the MR findings, and FDR-adjusted *P*-values were also calculated.

### Generalizability to East Asians and sex-specific analyses

To further assess the generalizability of our findings to East Asian populations, we used East Asian-specific summary statistics from two additional protein GWAS (*n*: 3965 and 3974) [27, 28], and a T2D GWAS (77 418 cases, 356 122 controls) [29]. We also explored whether the association of identified proteins with T2D varied by sex using sex-specific summary statistics from T2D GWAS [30].

### Sensitivity analyses

We conducted two sets of sensitivity analyses. First, to assess the robustness of our findings to the choice of proteomic platform, we replicated the analyses using protein associations from the UK Biobank Pharma Proteomics Project (UKB-PPP), which used the Olink platform [31]. Second, to test whether our Step 1 findings were sensitive to the definition of childhood adiposity, we repeated the analyses using an alternative exposure: self-perceived body size at age 10 from the UK Biobank (453 169 participants). This trait was derived from the question: ‘When you were 10 years old, compared to average would you describe yourself as thinner, plumper, or about average?’ [32]. Lastly, we checked whether the association of childhood BMI with identified proteins in the two-step MR is robust to outliers using MR-PRESSO.

### Enrichment analyses

Gene ontology (GO) and Kyoto Encyclopedia of Gene and Genome (KEGG) pathway enrichment analyses were performed separately for the six proteins identified as mediators by two-step MR, and for the eight protein aptamers (six unique proteins) associated with PRS_BMI_ in the recall-by-genotype study, to identify and compare the overrepresented functional categories and pathways. GO terms and KEGG pathways were considered significant at *P *< .05.

All analyses and visualization were achieved using R version 4.4.1 (R Foundation for Statistical Computing, Vienna, Austria) with the packages ‘*TwoSampleMR*’ (version 0.5.6) [33], ‘*coloc*’ (version 5.2.2) [20], ‘*clusterProfiler*’ (version 4.12.6) [34], and ‘*enrichplot*’ (version 1.24.4) [35].

## Results

### Association of genetically predicted childhood BMI with protein signatures

**Figure 2:**
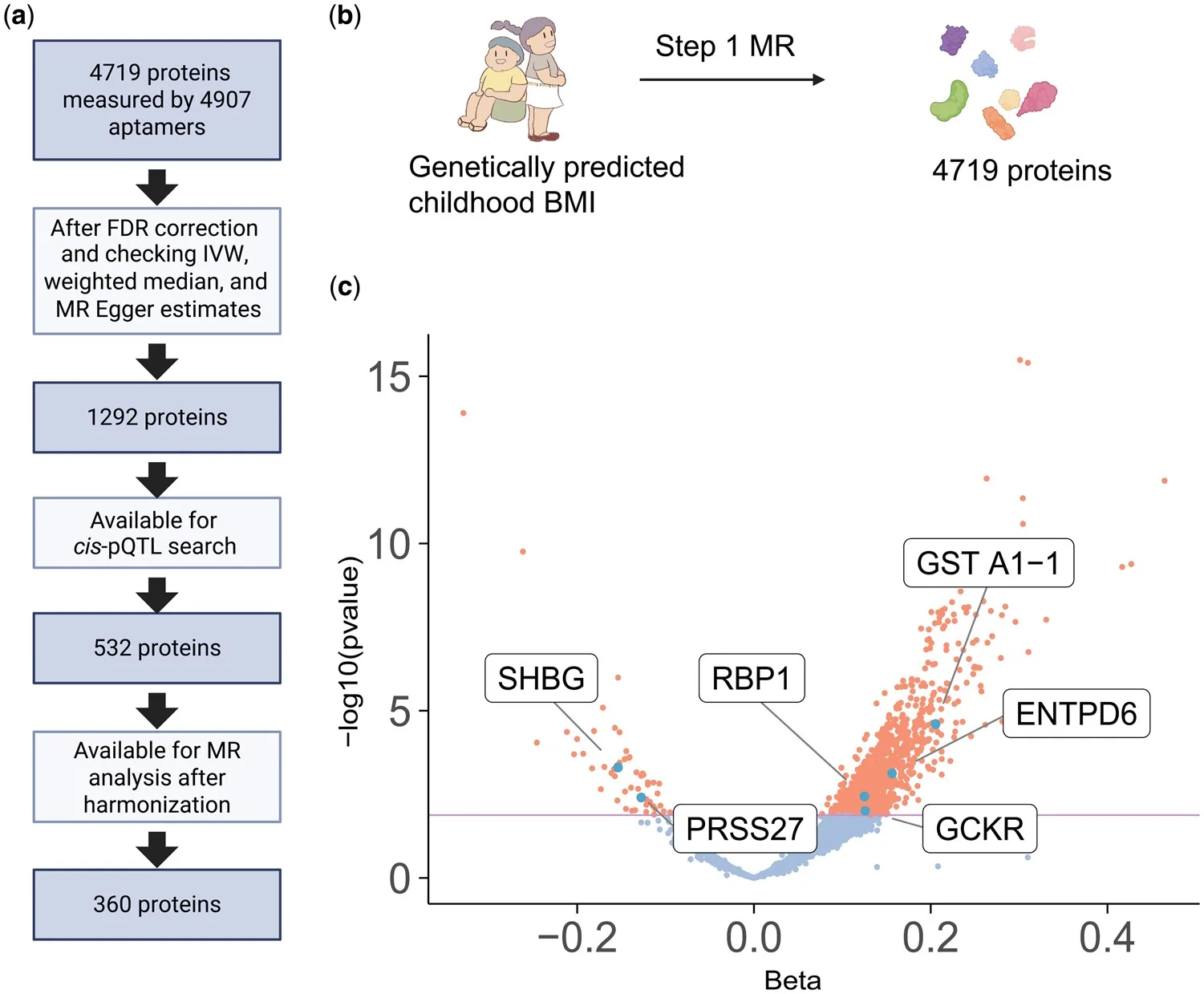
MR results for the effects of childhood BMI on 4719 plasma proteins (4907 aptamers). (a) Flow diagram of step 1 MR analyses. (b) Schematic diagram of step 1 MR analyses. (c) Volcano plot illustrating the effects of childhood BMI on 4719 plasma proteins (4907 aptamers) using inverse variance weighted method. The horizontal line indicates the FDR-adjusted significance threshold (corresponding to FDR-adjusted *P *= .05). Each point denotes a protein assessed. Protein elements were generated using BioRender. Created in Biorender. Chen, S. (2026) https://BioRender.com/lu8gtx3. FDR, false discovery rate; IVW, inverse variance weighted; cis-pQTL, cis-acting protein quantitative trait locus; MR, Mendelian randomization; BMI, body mass index; GCKR, glucokinase regulatory protein; RBP1, retinol-binding protein 1; ENTPD6, ectonucleoside triphosphate diphosphohydrolase 6; GSTA1-1, glutathione S-transferase A1-1; PRSS27, serine protease 27; SHBG, sex hormone-binding globulin.

Using 21 instruments for childhood BMI (mean F-statistic: 67, Supplementary Table S2), genetically predicted childhood BMI was associated with 1299 protein signatures (FDR-adjusted *P *< .05, Fig. 2 and Supplementary Table S3). The majority of these signals (1292 proteins) had consistent estimates from the IVW and weighted median. Despite signs of heterogeneity for 452 proteins (35%), evidence of horizontal pleiotropy was not evident for most proteins based on MR-Egger (2 proteins, 0.4%).

### Association of genetically predicted protein signatures with T2D risk

**Figure 3:**
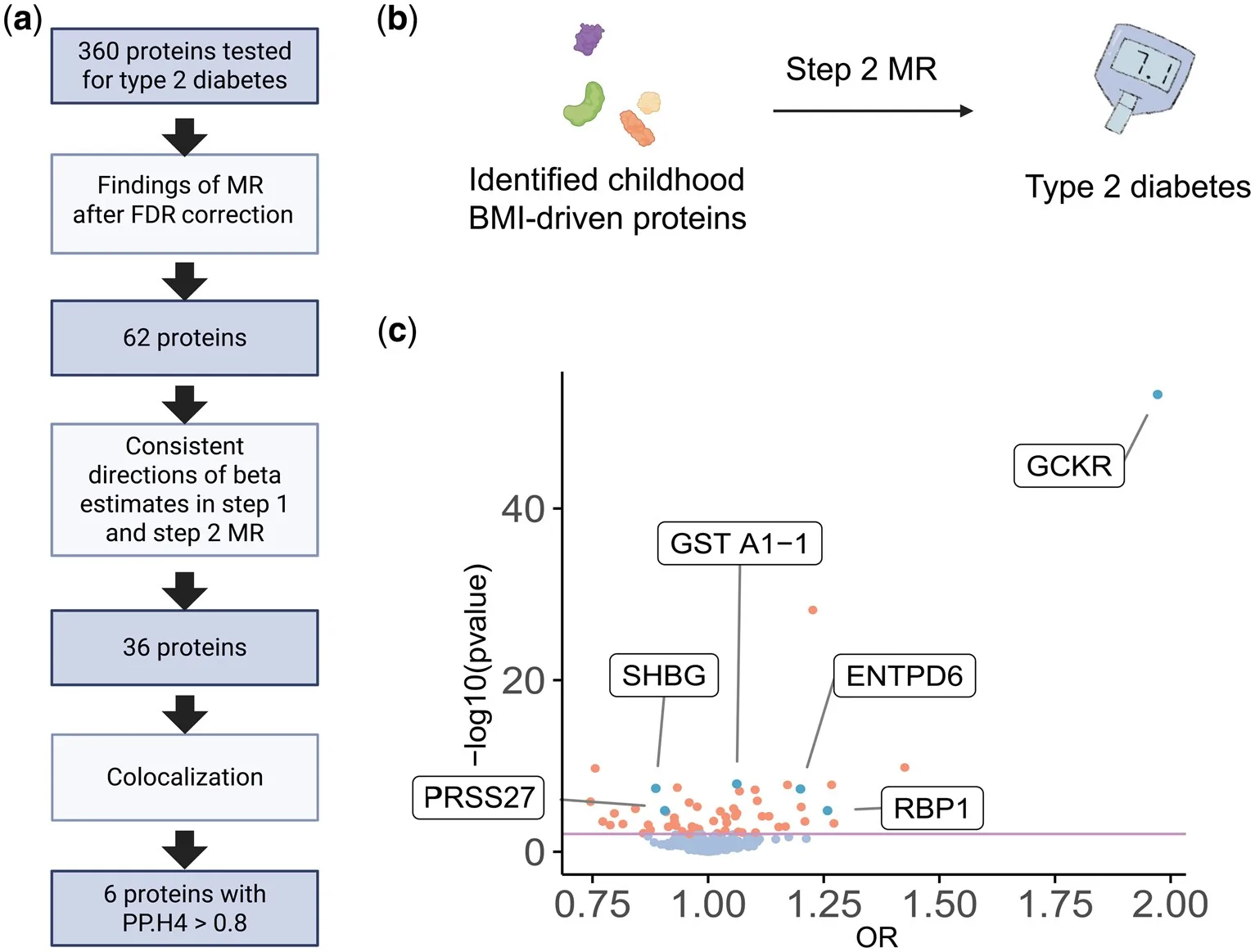
MR results for the effects of 360 identified proteins on T2D risk. (a) Flow diagram of step 2 MR analyses. (b) Schematic diagram of step 2 MR analyses. (c) Volcano plot illustrating the effects of 360 identified proteins using Wald ratio or inverse variance weighted method. The horizontal line indicates the FDR-adjusted significance threshold (corresponding to FDR-adjusted *P *= .05). Each point denotes a protein assessed. Protein elements were generated using BioRender. Created in BioRender. Chen, S. (2026) https://BioRender.com/v23gy52. MR, Mendelian randomization; FDR, false discovery rate; PP.H4, posterior probability of hypothesis 4 from colocalization analysis that two traits shared a single causal variant within a gene region; BMI, body mass index; OR, odds ratio; GCKR, glucokinase regulatory protein; RBP1, retinol-binding protein 1; ENTPD6, ectonucleoside triphosphate diphosphohydrolase 6; GSTA1-1, glutathione S-transferase A1-1; PRSS27, serine protease 27; SHBG, sex hormone-binding globulin.

Among 1292 proteins, 360 proteins (41.2%) had *cis*-pQTLs for downstream analyses (Supplementary Table S4). There are 62 genetically predicted proteins associated with T2D risk (FDR-adjusted *P *< .05) (Fig. 3 and Supplementary Table S5). After excluding associations with inconsistent directionality between Step 1 MR (childhood BMI to protein) and Step 2 MR (protein to T2D), given our hypothesis that childhood BMI increases T2D risk mediated by the proteins, 36 proteins were identified as plausible mediators (Supplementary Table S6).

### Colocalization

Pairwise colocalization analyses with default priors suggested that, amongst the 36 proteins, six showed strong evidence of colocalization with T2D (PP_H4_ > 80%). These included glucokinase regulatory protein (GCKR), retinol-binding protein 1 (RBP1), ectonucleoside triphosphate diphosphohydrolase 6 (ENTPD6), sex hormone-binding globulin (SHBG), serine protease 27 (PRSS27), and glutathione S-transferase A1 (GST A1-1). These findings were consistent across different prior combinations in the colocalization analyses and when using SuSiE (Supplementary Table S7 and Supplementary Figs S1–S6).

### Proportion of effect mediated

The proportion of the total effect of childhood BMI on T2D risk mediated by each protein is summarized in Supplementary Table S8. The mediated proportion ranged from 1.87% to 12.92%. GCKR mediated the largest proportion (12.92%, 95% confidence interval (CI): 1.87% to 23.96%), followed by RBP1 (4.33%, 95% CI: 0.46% to 8.21%) and ENTPD6 (4.29%, 95% CI: 0.95% to 7.63%). SHBG mediated 2.79% (95% CI: 0.66% to 4.92%), while PRSS27 and GST A1-1 mediated 1.89% (95% CI: 0.19% to 3.60%) and 1.87% (95% CI: 0.58% to 3.15%), respectively.

### Results from recall-by-genotype design in the Chinese birth cohort

In the RbG design using the Chinese birth cohort, only SHBG was replicated (*P *= .014). The other five proteins (GCKR, RBP1, ENTPD6, PRSS27, and GST A1-1) did not reach nominal significance (uncorrected *P* ranged from 0.48 to 0.88). However, differences in other protein markers between the highest and lowest PRS_BMI_ groups were observed for eight additional proteins, such as hyaluronan and proteoglycan link protein 1 (HPLN1), leptin, and osteomodulin (OMD) (FDR-adjusted *P *< .05, Supplementary Tables S9–S11).

### East Asian and sex-specific validation

East Asian-specific protein GWAS provided the summary statistics for four proteins (ENTPD6, GST A1-1, RBP1, and SHBG) (Supplementary Table S12). Replication analyses showed directionally consistent association between genetically predicted SHBG and T2D risk compared with the estimate observed in Europeans (Supplementary Table S13). Sex-specific analyses revealed no statistically significant differences in the effects of identified proteins on T2D risk between males and females (Wald test *P *> .05, Supplementary Tables S14 and S15).

### Sensitivity analyses

The UKB-PPP provided summary statistics for all identified proteins except GCKR. Only PRSS27 and SHBG were replicated in both MR and colocalization (Supplementary Tables S16 and S17). Associations between genetically predicted childhood BMI and the identified proteins were replicated with self-perceived body size at age 10 as an alternative exposure, with the exception of PRSS27 (Supplementary Tables S18 and S19). Associations between childhood BMI and the six identified proteins were robust to outliers based on MR-PRESSO (Supplementary Table S20).

Pathway enrichment analyses indicated no overlap between the significantly enriched terms in the two protein sets across biological process, molecular function, or cellular component domains; similar findings were observed using the KEGG database (Supplementary Figs S7–S10).

## Discussion

To the best of our knowledge, this is one of the first MR studies exploring the proteomic pathways linking childhood adiposity with T2D risk. Our findings indicate that two proteins, SHBG and GCKR, may mediate the effect of childhood BMI on T2D risk, with support from colocalization and sensitivity analyses. These proteins could potentially serve as clinical markers reflecting a prodiabetic status resulting from childhood adiposity. More importantly, they may represent putative intervention targets (e.g. gestrinone and levonorgestrel from Drugbank) to mitigate T2D risk arising from childhood adiposity. However, our RbG study in Chinese individuals suggests that many proteomic signatures may be more relevant to Europeans than to East Asians.

SHBG, a protein inversely associated with obesity, is a well-established protective factor against T2D [36]. Its testosterone-binding role introduces sex-specific risk modulation–higher free testosterone increases T2D risk in women while decreasing it in men [37]. In particular, the glucose-lowering allele of *GCKR* correlates with lower SHBG [36], suggesting a complex interplay between these two mediators. This underscores the need for sex-specific interventions, such as physical activity, which increases both SHBG and testosterone, targeting these pathways [38].

*GCKR* encodes a hepatic metabolic sensor that inhibits glucokinase, hence modulating glucose-to-triglyceride conversion [39]. The rs1260326-T allele (associated with reduced GCKR activity) lowers fasting glucose and T2D risk but elevates triglycerides, reflecting GCK-driven hepatic glycolysis and lipid storage [40]. While GCKR inhibition is a potential T2D target, its cardiometabolic trade-offs (hypertriglyceridemia) and paradoxical effects on β-cell function necessitate balanced strategies for T2D prevention in the general population [41].

A novel finding from this study is the distinctly different proteomic signatures related to childhood adiposity in Europeans and East Asians. Although the RbG study had a comparatively small sample size, it successfully replicated the expected higher leptin level in the upper extreme of PRS_BMI_ (*P*: 7.3 × 10^−6^) [42], and the SHBG signal. Notably, top hits from the recall-by-genotype study included HPLN1 (*P*: 9.7 × 10^−6^) and OMD (*P*: 2.5 × 10^−6^), which were not identified in the Europeans. How these proteins relate to T2D in East Asians remains unclear. However, a recent proteomic study showed lower OMD associated with higher T2D in Europeans [43], consistent with our observation that participants with higher PRS_BMI_ had lower OMD levels. The etiologic role of these proteins in T2D among East Asians requires further investigation.

The main strength of this study was the use of an MR design in Europeans combined with a RbG design in a Chinese birth cohort, enabling a comparison of proteomic signatures related to childhood adiposity across two ethnic groups. In particular, both designs are less vulnerable to confounding than conventional observational designs. Nevertheless, several limitations must be acknowledged. First, MR studies rely on stringent assumptions, although we implemented a series of sensitivity analyses and genetic colocalization analyses to rule out biases due to violations of these assumptions. Second, although we implemented the first RbG design in a Chinese birth cohort and provided less confounded results on ethnic differences in proteomic signatures, the lack of consistency between Europeans and East Asians may result from insufficient statistical power for proteins with small effect sizes or from overfitting in BMI instrument identification. Alternatively, differences in lifestyle, environmental, and cultural factors across ethnicity can also contribute to this heterogeneity. As such, these discrepancies require further verification. Third, our reliance on summary statistics limited our ability to explore potential nonlinear relations or sex-specific effects, the latter being particularly relevant given the substantially differing body composition of males and females. Fourth, we were not able to consider better proxies of adiposity [44], such as DXA-measured fat mass, due to the lack of corresponding GWAS. Similarly, self-reported body size is a proxy for BMI and may be vulnerable to recall bias [32]. Fifth, we did not account for adult BMI within a lifecourse MR framework. As childhood and adult adiposity are correlated, our estimates reflect the total effect of early-life BMI and do not distinguish direct effects from those operating via later-life BMI. Notably, previous lifecourse MR studies suggest that the association between childhood adiposity and T2D risk may be largely mediated through adult adiposity [32], indicating that some of the identified protein associations may reflect downstream effects of persistent adiposity rather than early-life-specific mechanisms. Lastly, while our study focused exclusively on proteomics, other intermediate markers, such as metabolites, could also be involved in the underlying mechanisms and should be considered in future studies.

In conclusion, our study highlights SHBG and GCKR as mediators of the effects of childhood adiposity on T2D risk in European populations. These proteins could serve as potential intervention targets to mitigate elevated T2D risk due to childhood obesity in the general population. However, the relevance of these findings to East Asian populations requires further investigation using ethnic-specific designs, such as MR in large-scale East Asian biobanks or other quasi-experimental designs.

## Ethics approval

The use of publicly available summary statistics did not require ethics approval, whereas ethics approval for individual GWAS can be found in corresponding publications [12, 13, 16, 32]. Ethical approval for the recall-by-genotype study based on ‘Children of 1997’ birth cohort was obtained from the Institutional Review Board of the University of Hong Kong/Hospital Authority Hong Kong West Cluster (HKU/HA HKW IRB) [24]. Informed written consent was confirmed and signed by the parents/guardians, or by the participant if they are 18 years or older before participation in the Biobank follow-up.

## Supplementary Material

dyag165_Supplementary_Data

## Data Availability

Data from the ‘Children of 1997’ birth cohort are available upon reasonable request from the ‘Children of 1997’ data access committee: aprmay97@hku.hk.
