# Supplementary material for: Elucidating the proteomic pathways linking childhood body mass index with type 2 diabetes: a Mendelian randomization and recall-by-genotype study
Source: Int J Epidemiol. 2026 Aug 18;55(5):dyag165. doi: 10.1093/ije/dyag165 (PMC13485351; doi:10.1093/ije/dyag165)
Supplement: Supplementary file 1 [file dyag165_supplementary_data.zip › ije-2025-11-2058-File006.docx]

**Supplementary Figures**

**Figure S1.** Regional association plot of GCKR protein and type 2 diabetes.

(Page 2)

**Figure S2.** Regional association plot of SHBG protein and type 2 diabetes. (Page 4)

**Figure S3.** Regional association plot of PRSS27 protein and type 2 diabetes. (Page 6)

**Figure S4.** Regional association plot of GSTA 1-1 protein and type 2 diabetes. (Page 8)

**Figure S5.** Regional association plot of RBP1 protein and type 2 diabetes. (Page 10)

**Figure S6.** Regional association plot of ENTDP6 protein and type 2 diabetes. (Page 12)

**Figure S7**. Bar plot of GO enrichment analysis results for the six proteins identified from the 2-step Mendelian randomization analyses and colocalization, i.e., GCKR, RBP1, ENTPD6, GST A1-1, PRSS27, and SHBG. (Page 14)

**Figure S8**. Bar plot of GO enrichment analysis results for the six proteins identified from the recall-by-genotype design, i.e., HPLN1, Leptin, OMD, CO9A1, CKLF4, and COL11A2. (Page 15)

**Figure S9**. Gene-concept network plot of KEGG enrichment analysis results for the six proteins identified from the 2-step Mendelian randomization analyses and colocalization, i.e., GCKR, RBP1, ENTPD6, GST A1-1, PRSS27 and SHBG. (Page 16)

**Figure S10**. Gene-concept network plot of KEGG enrichment analysis of the six proteins identified from the recall-by-genotype design, i.e., HPLN1, Leptin, OMD, CO9A1, CKLF4 and COL11A2. (Page 17)

**Figure S1.** Regional association plot of GCKR protein and type 2 diabetes.

Regional association plots show −log₁₀(P-values) from GWAS summary statistics for glucokinase regulatory protein (GCKR) protein levels (top panel) and type 2 diabetes (bottom panel) across a ±500 kb window centered on GCKR (around chr2: 27.2–28.2 Mb, GRCh38). Each dot represents a single SNP, with its vertical position indicating the association P-value on the left y-axis. The lead variant rs1260326 (*cis*-acting protein quantitative trait loci (cis-pQTLs) of GCKR) is highlighted as a purple diamond in both panels. Surrounding SNPs are coloured according to their linkage disequilibrium (r²) with rs1260326, estimated from the 1000 Genomes EUR reference panel. The light-blue line and right y-axis show the estimated recombination rate (cM/Mb) from HapMap. Gene annotations within the region are shown in the bottom track, with arrows indicating the direction of transcription. The shared lead signal and concordant LD pattern across both traits are consistent with colocalization at this locus (PP.H4 = 100% under *default* prior probabilities of 𝑝1 = 𝑝2 = 10^-4^ and 𝑝12 = 10^-5^).

**Figure S2.** Regional association plot of SHBG protein and type 2 diabetes.

Regional association plots show −log₁₀(P-values) from GWAS summary statistics for sex hormone-binding globulin (SHBG) protein levels (top panel) and type 2 diabetes (bottom panel) across a ±400 kb window centered on SHBG (around chr17: 7.1–7.9 Mb, GRCh38). Each dot represents a single SNP, with its vertical position indicating the association P-value on the left y-axis. In the top panel, the lead variant for SHBG, rs858519 (a cis-pQTL of SHBG), is highlighted as a purple diamond. In the bottom panel, the lead variant for type 2 diabetes, rs858518, is highlighted as a purple diamond; rs858519 is additionally labelled to indicate the position of the SHBG cis-pQTL within the type 2 diabetes association signal. Surrounding SNPs are coloured according to their linkage disequilibrium (r²) with the labelled lead variant, estimated from the 1000 Genomes EUR reference panel. The light-blue line and right y-axis show the estimated recombination rate (cM/Mb) from HapMap. Gene annotations within the region are shown in the bottom track, with arrows indicating the direction of transcription. rs858518 and rs858519 are in strong linkage disequilibrium (r² = 0.925 using EUR reference panel), indicating that both traits share the same underlying association signal. The concordant LD pattern across both traits is consistent with colocalization at this locus (PP.H4 = 85.4% under default prior probabilities of p₁ = p₂ = 10⁻⁴ and p₁₂ = 10⁻⁵).

**Figure S3.** Regional association plot of PRSS27 protein and type 2 diabetes.

Regional association plots show −log₁₀(P-values) from GWAS summary statistics for Serine protease 27 (PRSS27) protein levels (top panel) and type 2 diabetes (bottom panel) across a ±400 kb window centered on PRSS27 (around chr16: 2.37–3.2 Mb, GRCh38). Each dot represents a single SNP, with its vertical position indicating the association P-value on the left y-axis. The lead variant rs71386687 (a cis-pQTL of PRSS27) is highlighted as a purple diamond in both panels. Surrounding SNPs are coloured according to their linkage disequilibrium (r²) with rs71386687, estimated from the 1000 Genomes EUR reference panel. The light-blue line and right y-axis show the estimated recombination rate (cM/Mb) from HapMap. Gene annotations within the region are shown in the bottom track, with arrows indicating the direction of transcription. The shared lead signal and concordant LD pattern across both traits are consistent with colocalization at this locus (PP.H4 = 92.9% under default prior probabilities of p₁ = p₂ = 10⁻⁴ and p₁₂ = 10⁻⁵).

**Figure S4.** Regional association plot of GSTA 1-1 protein and type 2 diabetes.

Regional association plots show −log₁₀(P-values) from GWAS summary statistics for glutathione S-transferase A1 (GST A1-1) protein levels (top panel) and type 2 diabetes (bottom panel) across a ±400 kb window centered on rs2608631 (around chr6: 52.3–53 Mb, GRCh38). Each dot represents a single SNP, with its vertical position indicating the association P-value on the left y-axis. The lead cis-pQTL for GST A1-1, rs2608631, is highlighted as a purple diamond in both panels. Of note, the association P-value for rs2608631 in the GST A1-1 GWAS was numerically zero in the original summary statistics, with the accompanying −log₁₀(P) field reported as infinity (Inf) **(corresponding −log₁₀(***P***) is 429.2 in deCODE study’s supplementary table 2)**, indicating that the true P-value was smaller than the minimum positive value representable in double-precision floating-point arithmetic (~10⁻³⁰⁸). During instrument selection, the Mendelian randomization software applied a default lower bound of 1 × 10⁻²⁰⁰ to such extreme P-values, which is the value plotted here for rs2608631. Consequently, the purple diamond appears less significant than some neighbouring variants in the top panel, although the true association at rs2608631 substantially exceeds that of any other variant in the region. Surrounding SNPs are coloured according to their linkage disequilibrium (r²) with rs2608631, estimated from the 1000 Genomes EUR reference panel. The light-blue line and right y-axis show the estimated recombination rate (cM/Mb) from HapMap. Gene annotations within the region are shown in the bottom track, with arrows indicating the direction of transcription. The shared lead signal and concordant LD pattern across both traits are consistent with colocalization at this locus (PP.H4 = 97.6% under default prior probabilities of p₁ = p₂ = 10⁻⁴ and p₁₂ = 10⁻⁵).

**Figure S5.** Regional association plot of RBP1 protein and type 2 diabetes.

Regional association plots show −log₁₀(P-values) from GWAS summary statistics for retinol-binding protein 1 (RBP1) protein levels (top panel) and type 2 diabetes (bottom panel) across a ±400 kb window centered on rs2071387 (around chr3: 138.85–139.65 Mb, GRCh38). Each dot represents a single SNP, with its vertical position indicating the association P-value on the left y-axis. The lead cis-pQTL for RBP1, rs2071387, is highlighted as a purple diamond in both panels. Surrounding SNPs are coloured according to their linkage disequilibrium (r²) with rs2071387, estimated from the 1000 Genomes EUR reference panel. The light-blue line and right y-axis show the estimated recombination rate (cM/Mb) from HapMap. Gene annotations within the region are shown in the bottom track, with arrows indicating the direction of transcription. The shared lead signal and concordant LD pattern across both traits are consistent with colocalization at this locus (PP.H4 = 89.2% under default prior probabilities of p₁ = p₂ = 10⁻⁴ and p₁₂ = 10⁻⁵).

**Figure S6.** Regional association plot of ENTDP6 protein and type 2 diabetes.

Regional association plots show −log₁₀(P-values) from GWAS summary statistics for ectonucleoside triphosphate diphosphohydrolase 6 (ENTPD6) protein levels (top panel) and type 2 diabetes (bottom panel) across a ±400 kb window centered on rs6050446 (around chr20: 24.8–25.5 Mb, GRCh38). Each dot represents a single SNP, with its vertical position indicating the association P-value on the left y-axis. The lead cis-pQTL for ENTPD6, rs6050446, is highlighted as a purple diamond in both panels. Surrounding SNPs are coloured according to their linkage disequilibrium (r²) with rs6050446, estimated from the 1000 Genomes EUR reference panel. The light-blue line and right y-axis show the estimated recombination rate (cM/Mb) from HapMap. Gene annotations within the region are shown in the bottom track, with arrows indicating the direction of transcription. The shared lead signal and concordant LD pattern across both traits are consistent with colocalization at this locus (PP.H4 = 99.6% under default prior probabilities of p₁ = p₂ = 10⁻⁴ and p₁₂ = 10⁻⁵).

**Figure S7**. Bar plot of GO enrichment analysis results for the six proteins identified from the 2-step Mendelian randomization analyses and colocalization, i.e., GCKR, RBP1, ENTPD6, GST A1-1, PRSS27, and SHBG.

GO, Gene Ontology; CC, cellular component; MF, molecular function; GCKR, glucokinase regulatory protein; RBP1, retinol-binding protein 1; ENTPD6, ectonucleoside triphosphate diphosphohydrolase 6; GST A1-1, glutathione S-transferase A1; PRSS27, serine protease 27; SHBG, sex hormone-binding globulin.

**Figure S8**. Bar plot of GO enrichment analysis results for the six proteins identified from the recall-by-genotype design, i.e., HPLN1, Leptin, OMD, CO9A1, CKLF4, and COL11A2.

GO, Gene Ontology; CC, cellular component; MF, molecular function; HPLN1, hyaluronan and proteoglycan link protein 1; OMD, osteomodulin; CO9A1, collagen alpha-1(IX) chain; CKLF4, CKLF-like MARVEL transmembrane domain-containing protein 4; COL11A2, collagen alpha-2(XI) chain.

**Figure S9**. Gene-concept network plot of KEGG enrichment analysis results for the six proteins identified from the 2-step Mendelian randomization analyses and colocalization, i.e., GCKR, RBP1, ENTPD6, GST A1-1, PRSS27 and SHBG.

KEGG, Kyoto Encyclopedia of Genes and Genomes; GCKR, glucokinase regulatory protein; RBP1, retinol-binding protein 1; ENTPD6, ectonucleoside triphosphate diphosphohydrolase 6; GST A1-1, glutathione S-transferase A1; PRSS27, serine protease 27; SHBG, sex hormone-binding globulin.

**Figure S10**. Gene-concept network plot of KEGG enrichment analysis of the six proteins identified from the recall-by-genotype design, i.e., HPLN1, Leptin, OMD, CO9A1, CKLF4 and COL11A2.

KEGG, Kyoto Encyclopedia of Genes and Genomes; HPLN1, hyaluronan and proteoglycan link protein 1; OMD, osteomodulin; CO9A1, collagen alpha-1(IX) chain; CKLF4, CKLF-like MARVEL transmembrane domain-containing protein 4; COL11A2, collagen alpha-2(XI) chain.
